# Effectiveness of Mobile Health Interventions on Diabetes and Obesity Treatment and Management: Systematic Review of Systematic Reviews

**DOI:** 10.2196/15400

**Published:** 2020-04-28

**Authors:** Youfa Wang, Jungwon Min, Jacob Khuri, Hong Xue, Bo Xie, Leonard A Kaminsky, Lawrence J Cheskin

**Affiliations:** 1 Fisher Institute of Health and Well-Being College of Health Ball State University Muncie, IN United States; 2 Department of Nutrition and Health Sciences College of Health Ball State University Muncie, IN United States; 3 Department of Biomedical and Health Informatics Children’s Hospital of Philadelphia Philadelphia, PA United States; 4 School of Public Health Imperial College London London United Kingdom; 5 Department of Health Behavior and Policy Virginia Commonwealth University Richmond, VA United States; 6 School of Nursing The University of Texas at Austin Austin, TX United States; 7 School of Information The University of Texas at Austin Austin, TX United States; 8 Department of Nutrition and Food Studies George Mason University Fairfax, VA United States; 9 Department of Health Behavior and Society Johns Hopkins University Baltimore, MD United States

**Keywords:** diabetes mellitus, obesity, overweight, mHealth, mobile app, telemedicine

## Abstract

**Background:**

Diabetes and obesity have become epidemics and costly chronic diseases. The impact of mobile health (mHealth) interventions on diabetes and obesity management is promising; however, studies showed varied results in the efficacy of mHealth interventions.

**Objective:**

This review aimed to evaluate the effectiveness of mHealth interventions for diabetes and obesity treatment and management on the basis of evidence reported in reviews and meta-analyses and to provide recommendations for future interventions and research.

**Methods:**

We systematically searched the PubMed, IEEE Xplore Digital Library, and Cochrane databases for systematic reviews published between January 1, 2005, and October 1, 2019. We analyzed 17 reviews, which assessed 55,604 original intervention studies, that met the inclusion criteria. Of those, 6 reviews were included in our meta-analysis.

**Results:**

The reviews primarily focused on the use of mobile apps and text messaging and the self-monitoring and management function of mHealth programs in patients with diabetes and obesity. All reviews examined changes in biomarkers, and some reviews assessed treatment adherence (n=7) and health behaviors (n=9). Although the effectiveness of mHealth interventions varied widely by study, all reviews concluded that mHealth was a feasible option and had the potential for improving patient health when compared with standard care, especially for glycemic control (−0.3% to −0.5% greater reduction in hemoglobin A_1c_) and weight reduction (−1.0 kg to −2.4 kg body weight). Overall, the existing 6 meta-analysis studies showed pooled favorable effects of these mHealth interventions (−0.79, 95% CI −1.17 to −0.42; I2=90.5).

**Conclusions:**

mHealth interventions are promising, but there is limited evidence about their effectiveness in glycemic control and weight reduction. Future research to develop evidence-based mHealth strategies should use valid measures and rigorous study designs. To enhance the effectiveness of mHealth interventions, future studies are warranted for the optimal formats and the frequency of contacting patients, better tailoring of messages, and enhancing usability, which places a greater emphasis on maintaining effectiveness over time.

## Introduction

### Background

Diabetes and obesity have become global epidemics [1,2]. The global prevalence of type 2 diabetes mellitus (T2DM) and overweight/obesity among adults had increased from 9% in 2014 [3] to 40% in 2016 [4]. They both have significant and overlapping health and economic consequences, such as excess morbidity and mortality and health care resource utilization [5,6]. The global direct cost of diabetes and obesity has been estimated at US $825 billion and US $2 trillion per year, respectively [7,8]. However, patients often do not have adequate access to or cannot afford health care. Many others are not able to adhere to their treatment regimen, particularly in low-resource settings [9]. These diseases are difficult to manage effectively, and thus, patients suffer from more complications, in addition to the financial burden on themselves and society [9,10]. Thus, providing adequate health care services that enable patients to manage these chronic diseases is critical.

Self-management practices, such as maintaining a healthy diet and weight, engaging in adequate physical activity (PA), using prescribed medications consistently, frequently checking body weight and blood sugar levels, and maintaining good mental health habits, help patients control diabetes and obesity efficiently [11]. Previous studies have shown that self-management support in diabetes improves hemoglobin A_1c_ (HbA_1c_) levels, reduces risks of developing life-threatening complications, and positively affects patient psychosocial and behavioral health [6]. However, the lack of individualized and coordinated care, inconvenient and costly education programs, and poor patient-provider communication make self-management practices challenging to adhere to and maintain. Effective services and methods for self-management are needed to reduce health care costs associated with these conditions, while improving the patient’s quality of life [12].

Emerging mobile health (mHealth) approaches may help meet these needs. In both developed and developing countries, mobile technology and device usage has been rapidly increasing and plays a vital role in people’s daily life [13]. Mobile technology provides mobility, instant access, and direct communication, which allows for faster transfer of health information and efficient health management assistance for patients [14,15]. It can also help provide better and expanded access to more affordable health services in low-income countries and low socioeconomic status groups in middle- and high-income countries [16]. Mobile technologies, specifically mobile apps, present an opportunity to help patients improve their adherence to health care providers’ advice, enhance patient-provider communication, and help facilitate and maintain behavioral changes [17,18]. mHealth is increasingly being used to improve the access and delivery of health services, treatment adherence, and management of various diseases and health risk–altering behaviors, such as HIV/AIDS, malaria, tuberculosis, diabetes, asthma, obesity, and smoking [16]. However, research on the applications of mHealth is still at an early stage of development and translation, and many unanswered questions remain.

The availability of commercial chronic disease self-management apps has been increasing rapidly [19,20]. Commercial apps may offer patients many high-quality choices in the self-management of their diseases and conditions. However, the large number of these apps makes it difficult for patients and health care providers to choose among the options wisely. Furthermore, only a small proportion of apps, other mobile devices, and programs have been appropriately tested for effectiveness.

Some previous reviews, including ours, have described the development of app technologies and their utility for patients with obesity, diabetes, and other chronic conditions [19,21-37]. However, their scope did not adequately address the effectiveness of mHealth for diabetes and obesity treatment and management. Many reviews have concluded that mHealth is promising for disease control but reported inconsistent findings on its effectiveness. Furthermore, the methods used in previous reviews have often been flawed for reasons such as not providing quantitative results, conducting a quantitative analysis with clinical/nonclinical trials and other study designs together, not using standardized data extraction, and a limited scope of review [24,35]. A thorough review of the evidence is needed and can help guide future research and interventions [38,39].

### Objectives

This study evaluated the effectiveness of mHealth interventions for diabetes and obesity treatment/management by examining published systematic reviews and meta-analyses and provided recommendations for future research and interventions.

## Methods

### Study Selection

#### Database and Literature Search Strategy

We searched the PubMed, IEEE Xplore Digital Library, and Cochrane databases to identify systematic reviews and meta-analyses published in English between January 1, 2005, and October 1, 2019, that evaluated the effectiveness of mHealth interventions for obesity and/or diabetes treatment/management. For the search, combinations of key terms were used in the PubMed, for example, “mhealth[Title/Abstract] AND (obesity[Title/Abstract] OR diabetes*[Title/Abstract]) AND review[Title/Abstract].” Search results were further screened manually by study title, abstract, and full text on the basis of inclusion and exclusion criteria.

**Figure 1:**
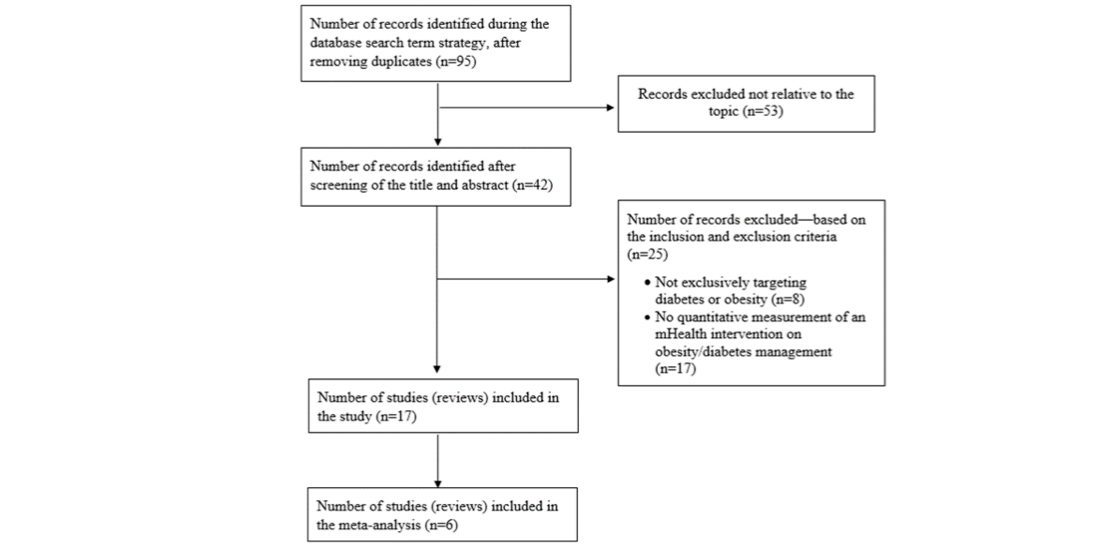
A flow chart of the literature search and study selection procedures. mHealth: mobile health.

The initial search yielded 95 articles. After eliminating duplicates and studies that did not fit the inclusion criteria, 17 reviews meeting the inclusion criteria remained; 6 of the 17 reviews were meta-analyses with randomized controlled trials (RCTs; Figure 1). The 17 reviews assessed 55,604 original intervention studies.

#### Study Inclusion Criteria

Studies were included if they (1) reviewed intervention studies on patients with obesity or/and diabetes; (2) were a systematic review and/or a meta-analysis; (3) tested an mHealth intervention (eg, use of mobile devices, apps, and text message) for managing or treating obesity/diabetes while measuring clinical biomarkers, treatment adherence, or health-related behaviors (eg, healthy eating and exercise); and (4) provided quantitative results examining the effectiveness of the intervention (or use of the mHealth devices/programs).

#### Study Exclusion Criteria

Studies were excluded if they (1) did not explicitly target diabetes or obesity; (2) were diabetes or obesity prevention studies, not using an mHealth-based intervention program; and (3) did not report quantitative outcomes of mHealth intervention effects in managing obesity or diabetes.

#### Study Quality Assessment

We used the Assessment of Multiple Systematic Reviews (AMSTAR 2) to assess the quality of selected studies by 16 criteria (eg, study selection, data extraction, assessing risk of bias, study description, and statistical methods) according to the study characteristics [40]. We assigned 1 point to each item that scored *yes* and summed these to calculate a total score (ranging from 0 to 16) for each review. We classified the quality of systematic reviews as high (score range 12-16), moderate (score range 9-11), low (score range 5-8), or critically low (score range 0-4; Multimedia Appendix 1) [41].

### Data Extraction and Statistical Analysis

Data were reviewed and extracted by 2 coauthors following the Preferred Reporting Items for Systematic Review and Meta-Analysis guidelines [42]. The information extracted included study year; design; objective; literature search scope and date; the number of articles accessed and included in the systematic review; and nature of the intervention, such as application type and targeted function, outcome measures related to clinical biomarkers, treatment adherence and health-related behaviors, and effectiveness of the mHealth intervention.

Using mixed effect models, we conducted a meta-analysis to evaluate the overall effectiveness of mHealth interventions on the basis of other published meta-analysis results with RCTs. The STATA (StataCorp LLC) metan command was used to calculate pooled estimates of mean differences in changes in clinical outcomes such as HbA_1c_, body weight, and BMI between intervention and control groups [21-23,32-34].

## Results

### Main Characteristics of the 17 Included Reviews

#### Study Topics and Study Design

Multimedia Appendix 2 describes the characteristics of the 17 reviews: 7 studies were conducted in the United States and the others were conducted in Australia (n=1), Canada (n=1), China (n=3), Germany (n=1), New Zealand (n=1), South Korea (n=1), Spain (n=1), and the United Kingdom (n=1). Regarding study design, 15 of the reviews were based on clinical trials, of which 9 included only RCTs, whereas 6 also included quasi-experimental studies. One review was rated as critically low quality (AMSTAR 2 score 0-4), 5 were rated as low quality (AMSTAR 2 score 5-8), 5 were rated as moderate quality (AMSTAR 2 score 9-11), and 6 were categorized as high quality (AMSTAR 2 score 12-16). Of the 17 reviews, 10 reviewed mHealth interventions in patients with diabetes, 6 in patients with overweight/obesity, and 1 included both conditions. Meta-analyses for various health outcomes were conducted in 6 reviews with RCTs.

We conducted the meta-analysis of the *mean difference* on the basis of the results reported in the 6 reviews using a mixed effect model. The STATA metan command was used to calculate pooled estimates, with confidence limits of mean difference in clinical outcomes. The I^2^ statistic quantifies the percentage of variability that can be attributed to between-study differences.

The mean difference was calculated by subtracting the level of clinical outcomes at the end of follow-up from the baseline, comparing the intervention and control groups. This allowed for a comparison of clinical improvement because of the mHealth interventions vs the control group.

#### Types of Mobile Health Interventions

We categorized the mHealth interventions studied into 5 types (Multimedia Appendix 2): (1) an app, which uses smartphones to deliver educational materials or help patients self-manage their health condition; (2) web-based tools used to provide patient education and/or advice on self-management; (3) text messaging, which uses mobile phone text messages as the primary mode of communication between patients and health care providers; (4) a portable monitoring device/personal digital assistant (PDA), which typically offers patient data collection over a wireless connection and can monitor patients’ physiological status; and (5) a pedometer that counts the number of steps taken in a day. These classifications were made on the basis of several considerations, including simplicity, understandability for a nontechnical audience, and the technological complexity involved in the intervention [31].

Mobile apps were the most widely studied intervention type (15 reviews), followed by text messaging (11 reviews) and PDAs (5 reviews). Regarding the major targeted functions of the mHealth interventions reviewed, self-monitoring and management was most common (15 reviews), followed by education or health promotion (8 reviews), reminders or alerts (5 reviews), feedback (3 reviews), social or peer support (2 reviews), and counseling or entertainment (1 review; see Multimedia Appendices 2 and 3).

#### Targeted Outcomes

All 17 reviews examined changes in clinical biomarkers as outcomes, whereas 9 evaluated health-related behaviors and 7 assessed treatment adherence (Multimedia Appendix 2). HbA_1c_ levels were included as clinical biomarkers in all the reviews of diabetes, whereas body weight/weight status (6 reviews) and BMI (5 reviews) were the main outcomes for obesity intervention reviews. Blood pressure (4 reviews), serum lipid/cholesterol levels (4 reviews), waist circumference, severe hypoglycemia/adverse effects (1 review), and C-reactive protein level (1 review) were also explored. For measuring treatment adherence, medication/treatment adherence and glycemic self-control/monitoring were most common. As indicators of health-related behaviors, PA and diet were frequently measured (n=6), whereas PA alone (n=1), other obesity-related behaviors, or self-care behaviors were less frequently reviewed (n=2).

### The Effectiveness of Mobile Health Interventions in Managing Obesity or Diabetes

#### Clinical Outcomes

We found much heterogeneity in the effectiveness of mHealth interventions for clinical biomarkers (Multimedia Appendix 3). For blood glucose control (including HbA_1c_), 8 reviews reported statistically significant or large improvements (more than or equal to half of the included studies) [21-23,25,26,29-31], although another 3 reported low improvements (less than half of the included articles) [24,27,28]. Treatment effects on BMI, weight, and waist circumference varied; 5 reviews found large improvements [31-35] and 3 reported small or no effect of the mHealth interventions [23,36,37]. mHealth interventions were found ineffective for improving serum lipids changes in 2 reviews [23,28], whereas 1 review found a few positive changes in cholesterol levels [29]. Blood pressure levels showed small improvements in 2 reviews [24,29], but 2 other reviews found no effect [23,28]. The heterogeneous results may reflect differences in study subjects (eg, T2DM vs type 1 diabetes mellitus [T1DM]) [21,30] and the severity of symptoms (HbA_1c_<8% vs HbA_1c_≥8%) [23]. The small number of reviews on serum lipids, cholesterol, and blood pressure (eg, <5 reviews) may be insufficient to examine the effectiveness of mHealth for these parameters.

**Figure 2:**
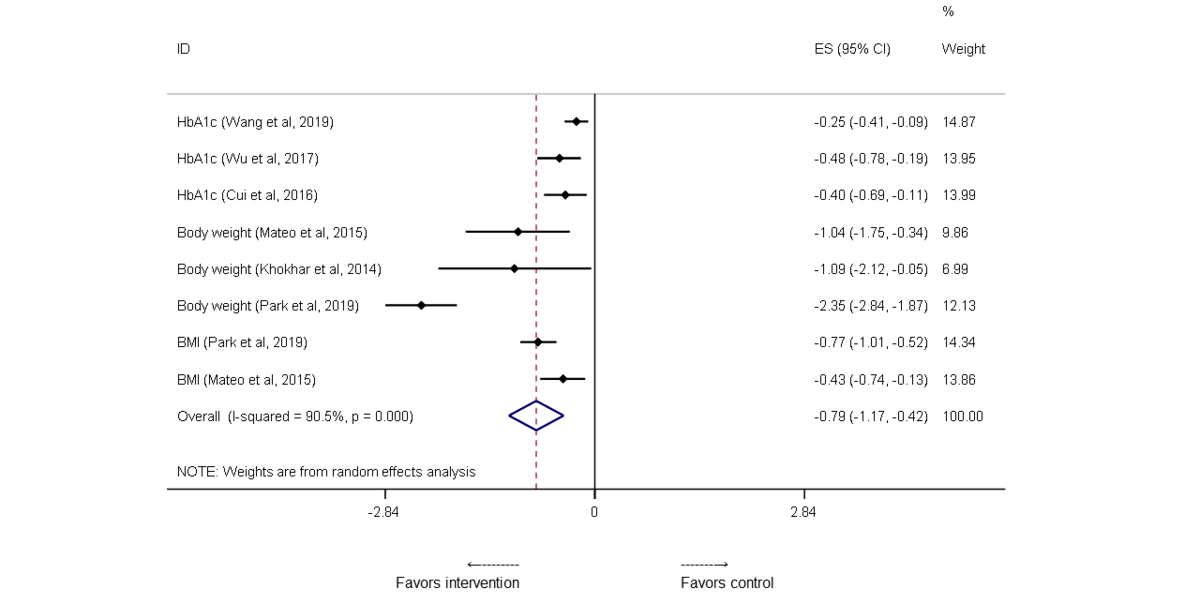
A meta-analysis of mean differences in changes in clinical outcomes after an intervention, mobile health versus control groups. HbA_1c_: hemoglobin A_1c_.

Regarding the meta-analyses, 3 reviews reported on the effect of mobile apps on HbA_1c_ levels in diabetes [21-23]. A meta-analysis indicated a significant reduction in HbA_1c_ from 0.25% (95% CI −0.41 to −0.09) [21] to 0.48% (95% CI −0.78 to −0.19) [22], presented in Table 1 and Figure 2, but with substantial heterogeneity in the pooled effect (I^2^ up to 77%). In particular, differences between the mHealth intervention group and the control group were significant for patients with HbA_1c_ <8% at baseline by −0.33% (−3.61 mmol/mol; I^2^= 70%), whereas it was not significant in the patients with HbA_1c_ ≥8% (*P*=.33) [23]. In addition, larger reductions were noticed after app use in HbA_1c_ among patients with T2DM (−0.67%, 95% CI −1.03 to −0.30) [22] compared with patients with T1DM (−0.37%, 95% CI −0.86 to −0.12).

A meta-analysis on RCTs consistently found that app use was associated with significant improvements in body weight and BMI [32-34] from −1.04 kg (95% CI −1.75 to −0.34; I^2^=41%) [33] to −2.35 kg (95% CI −2.84 to −1.87; I^2^=94%) [32] and from −0.43 kg/m^2^ (95% CI −0.74 to −0.13; I^2^=50%) [33] to −0.77 kg/m^2^ (95% CI −1.01 to −0.52; I^2^=0%) [32] than the control group, respectively. When stratified by the application type, only mobile-based interventions showed significant body weight loss (−1.78 kg, 95% CI −2.92 to −0.63; I^2^=16%), whereas PDA-based interventions showed nonsignificant changes (−0.23 kg, 95% CI −0.87 to 0.41; I^2^=0.0%) [34].

**Table 1:** Summary of clinical outcomes and behavioral changes from 18 meta-analyses reported in 6 reviews of diabetes and obesity mobile health interventions.

Outcomes	References^a^	Tested interventions/target patient	Intervention vs control groups	Estimated effect of intervention: meta-analysis results of the mean difference between intervention and control groups	Conclusions
HbA_1c_^b^	Wang et al [21]	Self-management of patients with T1DM^c^	Mobile app or text messaging intervention vs standard care	−0.25% (95% CI −0.41 to −0.09; I^2^=12%) Subgroup analysis—age: teenagers −0.05% (95% CI −0.43 to 0.33; I^2^=0%); adults −0.29% (95% CI −0.47 to −0.11; I^2^=48%) Subgroup analysis—intervention: text message −0.20% (95% CI −0.73 to 0.32; I^2^=0%); mobile apps −0.25% (95% CI −0.42 to −0.08; I^2^=49%) Subgroup analysis—duration: ≥6 months −0.29% (95% CI −0.46 to −0.11; I^2^=32%); <6 months −0.01% (95% CI −0.44 to 0.41; I^2^=0%)	mHealth^d^ favors
HbA_1c_	Wu et al [22]	Self-management of patients with diabetes	Mobile app intervention vs standard care alone	−0.48% (95% CI −0.78 to −0.19; I^2^=76%) Subgroup analysis: patients with T2DM^e^ −0.67% (95% CI −1.03 to −0.30; I^2^=47%); patients with T1DM −0.37% (95% CI −0.86 to −0.12; I^2^=86%)	mHealth favors
HbA_1c_	Cui et al [23]	Self-management of patients with T2DM	Smartphone app strategies vs standard diabetes care	−0.40% (95% CI −0.69 to −0.11; I^2^=77%) Subgroup analysis: baseline HbA_1c_<8% −0.33% (95% CI −0.59 to −0.06; I^2^=70%)	mHealth favors
Body weight	Park et al [32]	Weight loss interventions on patients with OWB^f^	Mobile app/text messaging intervention vs nonmobile device care (standard)	−2.35 kg (95% CI −2.84 to −1.87; I^2^=94%) Subgroup analysis—duration: at 6 months −2.66 kg (95% CI −3.94 to −1.38; I^2^=95%); at ≥12 months −1.23 kg (95% CI −2.25 to −0.21; I^2^=0%)	mHealth favors
Body weight	Mateo et al [33]	Weight loss and PA^g^ promotion on patients with OWB	Mobile app intervention vs the control diet	−1.04 kg (95% CI −1.75 to −0.34; I^2^=41%)	mHealth favors
Body weight	Khokhar et al [34]	Weight loss interventions on patients with OWB	Mobile electronic device intervention vs the control	−1.09 kg (95% CI −2.12 to −0.05; I^2^=50%) Subgroup analysis—duration: ≤6 months −0.97 kg (95% CI −2.23 to 0.30; I^2^=47%); >6 months −1.20 kg (95% CI −3.34 to 0.94; I^2^=62%) Subgroup analysis—intervention: mobile phone −1.78 kg (95% CI −2.92 to −0.63; I^2^=16%); personal digital assistant −0.23 kg (95% CI −0.87 to 0.41; I^2^=0.0%)	mHealth favors
BMI	Park et al [32]	Weight loss interventions on patients with OWB	Mobile app/text messaging intervention vs nonmobile device care (standard)	–0.77 kg/m^2^ (95% CI −1.01 to −0.52; I^2^=0%) Subgroup analysis—duration: at 3 months −1.10 kg/m^2^ (95% CI −2.79 to 0.59; I^2^=95%); at 6 months −0.67 kg/m^2^ (95% CI −0.71 to −0.63; I^2^=0%)	mHealth favors
BMI	Mateo et al [33]	Weight loss and PA promotion on patients with OWB	Mobile app intervention vs the control diet	−0.43 kg/m^2^ (95% CI −0.74 to −0.13; I^2^=50%)	mHealth favors
Physical activity	Mateo et al [33]	Weight loss and PA promotion on patients with OWB	Mobile app intervention vs control intervention	Standardized mean difference in net change 0.40 (95% CI −0.07 to 0.87; I^2^=93%)	No significant difference

^a^We selected 6 meta-analyses on randomized controlled trial studies. Please see our pooled meta-analysis presented in Figure 2.

^b^HbA_1c_: hemoglobin A_1c_ (glycated hemoglobin).

^c^T1DM: type 1 diabetes mellitus.

^d^mHealth: mobile health.

^e^T2DM: type 2 diabetes mellitus.

^f^OWB: overweight and obesity.

^g^PA: physical activity.

#### Treatment Adherence

Relatively few reviews examined the treatment effect of mHealth interventions and reported inconsistent results. Out of the 7 reviews that investigated mHealth intervention effects on treatment adherence, 4 reviews found a moderate improvement in glycemic control [23,26], greater adherence in the mHealth intervention group compared with the control group [35], or a reduced attrition rate during an obesity intervention program [37]. However, the other 2 reviews found that fewer than half of the studies improved diabetes management practices [24,28] or medication adherence [28].

#### Behavioral Changes

Results for behavioral changes were not consistent, and 3 diabetes reviews [24,26,28] and 1 obesity review [35] found a slight improvement in patients’ diets, eating habits, and PA behaviors. However, 3 reviews found inconsistent behavioral changes by disease (diabetes vs obesity) [21] or type of behavior (eating habits/dietary intake vs PA) [36,37], and 1 review found no significant effect of mHealth interventions on the level of PA [33]. A meta-analysis of RCTs found a nonsignificant difference in PA between the intervention and control groups, with a standardized mean difference of 0.40 (95% CI −0.07 to 0.87; I^2^=93%; Table 1).

Our meta-analysis (see Figure 2) on results from the existing 6 meta-analysis studies on HbA_1c_, body weight, and BMI shows an overall effect of −0.79 (95% CI −1.17 to −0.42; I^2^=90.5), which shows a pooled favorable effect of these mHealth interventions.

## Discussion

### Principal Findings

Although there is a strong interest among researchers, health care workers, and patients in mHealth interventions for the treatment of diabetes and obesity, overall, very little is known about its effectiveness. Moreover, at present, the use of mHealth interventions for these conditions is limited. To our knowledge, this is the first study that provides a comprehensive summary of research assessing the effectiveness of mHealth interventions for these conditions.

Published research has yielded mixed results. Examining evidence reported in 17 reviews that assessed a total of 55,604 original studies, this systematic review found that, overall, the impact of mHealth interventions on diabetes and obesity management is promising, especially in the areas of glycemic control and weight management. The majority of the 17 reviews focused on the self-monitoring functions of mHealth. Text messaging and apps were the primary types of mHealth interventions utilized to date. There was heterogeneity in the effectiveness of mHealth as diverse health outcomes (eg, blood pressure, weight, lipids, HbA_1c_, clinical biomarkers, treatment adherence, and health-related behavior changes) were tested in the original studies, but only a few studies with various study designs and populations (eg, clinical trials, nonclinical trials, and diverse patient subgroups by severity and disease type) and study focus (eg, incentive-driven technology) were available in the review. Nevertheless, all the 17 reviews concluded that mHealth was feasible and potentially can improve health outcomes among patients suffering from diabetes and/or obesity.

### Sources of Variations in Existing Research

Clinical biomarkers such as glycemic control and weight change were the primary focus in evaluating the effect of mHealth interventions in the reviews assessed. For example, the change in HbA_1c_ pre- and postintervention was evaluated in 10 reviews. Of these, 7 reviews reported statistically significant/large improvements, but 3 reviews did not; 2 meta-analyses showed 0.25% to 0.48% greater changes in HbA_1c_ following an mHealth intervention compared with standard diabetes care. In contrast, only 4 reviews found some improvement in treatment adherence in all 7 reviews that assessed it. Furthermore, small or insignificant improvements in health-related behaviors were reported in 9 reviews.

Several factors could have caused substantial heterogeneity among the assessment of clinical biomarkers, treatment adherence, and health-related behaviors. First, a small number of original studies examining treatment adherence and behavioral changes might have underpowered the systematic approach in the literature review. Second, the inclusion criteria for the study design (eg, clinical/nonclinical trial and quasi-experimental study), study subjects (eg, mixture of patients with T1DM and T2DM, patients with T1DM only, or poorly controlled patients with diabetes), and application type were not controlled efficiently in the previous reviews. In addition, patient health–related behaviors may require more time to change than was generally allowed in the studies compared with the typically more rapid change in biomarkers, possibly because of the influence of cognitive biases, habits, and social behavioral norms [43].

### Implementation and Dissemination of Mobile Health Interventions

In recent years, there has been explosive growth in the number of mobile apps [13,44], including mHealth apps. However, the large number of available mHealth apps may hinder the intended use of these apps [20,27,45]. Limited guidance and the commerce-influenced nature of internet-based searches make it difficult for patients to determine which apps could most effectively help manage their health conditions. The number of app functions has also been negatively correlated with user ratings [46]. Thus, a process of a truly objective app review has the potential to improve the ability of patients to find the appropriate apps that meet their needs and preferences [47]. In addition, this can also help health care providers in making clear recommendations of the best apps to use.

As education and health promotion can favorably influence clinical outcomes, app developers need to fully consider the needs of users in designing features for patients suffering from diabetes/obesity. For example, self-management should be promoted as a key feature in apps targeting patients with T1DM who may need to check their blood glucose level more frequently than those with T2DM. In addition, new mobile messaging services, such as Facebook Messenger, WhatsApp, Snapchat, and Instagram, now exceed the functionality of traditional text messaging. Relevantly, social media features are increasingly popular, particularly among young people. Social networks can help patients achieve behavioral changes by, for instance, providing peer support among patients with similar conditions [48]. Strategies targeting behavioral changes to enhance self-management for patients are not very common among existing apps [49-52]. Thus, mHealth apps could implement social media and network features to more effectively target young users and improve their care [53,54]. Of course, support and adoption of mHealth approaches for the treatment of diabetes and obesity by health care providers will be helpful in maximizing the potential future value of mHealth interventions.

### Limitations of Previous Reviews and the Original Mobile Health Intervention Studies

The 17 reviews and the included intervention studies share some limitations. First, some reviews only included a small number of studies but examined a relatively large number of outcomes [23,27-29]. Second, most of the reviews examined studies conducted in developed countries; few reviews examined those from developing countries [21,28,31]. Third, a heterogeneous study design may cause substantial heterogeneity in meta-analyses or make it difficult to conduct a quantitative analysis across studies [23,28-31,33,37]. Fourth, the reviews included only a small number of RCTs [27,28,31,33,34], and many of the RCTs had short intervention periods [29,31,33,37]. Fifth, only a limited number of original studies reported changes in biomarkers, which may hinder the evaluation of mHealth interventions’ clinical impact, especially on blood lipids and blood pressure [23,24]. Finally, the diverse features of the actual mHealth interventions further increase study heterogeneity [24].

### Limitations of This Study

First, we examined the results reported in the 17 identified reviews without analyzing the findings from the original studies. Second, there was a high level of heterogeneity in the characteristics and findings of the 17 reviews. Thus, it was challenging to adequately interpret the effectiveness of mHealth interventions across reviews because of different study designs, objectives, and settings. Despite these limitations, this study provided a higher level of analysis and a comprehensive summary of the findings in the growing mHealth field. Compared with previous studies, our study has a number of unique contributions, including the following: (1) our study added quantitative evidence specifically on the applications of mHealth in diabetic and obesity care research and studied objective changes in biomarkers, treatment adherence, and health behaviors after an mHealth intervention, whereas previous studies were general and narratively described mHealth effects on diverse diseases using a small number of articles with low quality; (2) we conducted a meta-analysis on the intervention effects of clinical outcomes, which was lacking in the existing reviews; and (3) our review included newly published reviews that were not included in other studies. This helps identify best practices for fighting the epidemics of diabetes and obesity. In addition, we found a fairly consistent reduction in HbA_1c_ and body weight from mHealth interventions across multiple reviews.

### Recommendations for Future Research

Regarding future evaluations of mHealth interventions, more rigorous study designs and strategies are needed to enable us to draw more precise and specific conclusions regarding their effectiveness for diabetes and obesity management. To enhance app design, including user ratings and experiences may be useful in developing evidence-based strategies. The level to which users truly engage with these mHealth apps is not yet clear. Patient-centered self-monitoring with personalized feedback is important in behavioral change and has been shown to improve user engagement and adherence [55]. Designing app functions relevant to the users on the basis of their age and sex, type of diabetes, and geographical location would improve the targeting and effectiveness of mHealth interventions.

To promote an evidence-based approach in mHealth use for diabetes and obesity management, multiple validation tests and, when appropriate, regulations will be needed. Objective and validated measures should be used, in particular, when studying behavioral changes following mHealth interventions. Furthermore, there is a need to identify and focus on high-risk groups (eg, low socioeconomic status populations), as most previous reviews did not include studies conducted in these populations.

In conclusion, findings from the 17 reviews, including 6 meta-analyses published since 2005, suggested promising but limited evidence on the effectiveness of mHealth interventions for diabetes and obesity management. Self-management, monitoring, and use of text messaging and apps are the primary target functions and application types of mHealth investigated in the field. More rigorous study designs should be applied in future studies for assessing the impact of mHealth interventions on diabetes and obesity management. To enhance the effectiveness of mHealth interventions, studies are warranted for the optimal formats and the frequency of contacting patients, using theory-based interventions; for the better tailoring of messages to the specific needs and communication style of recipients; and for enhancing the usability by adapting approaches to recipients with varying degrees of technological and health literacy, thus placing a greater emphasis on maintaining effectiveness over time.

## Appendix

Multimedia Appendix 1 Methodological quality of 17 studies based on AMSTAR2 criteria.

Multimedia Appendix 2 Characteristics of 17 review studies on the effectiveness of mobile health interventions for diabetes and obesity management.

Multimedia Appendix 3 Summary of findings from the 17 reviews on the effectiveness of mobile health interventions for diabetes and obesity management.

## Abbreviations

HbA_1c_: hemoglobin A_1c_
mHealth: mobile health
PA: physical activity
PDA: personal digital assistant
RCT: randomized controlled trial
T1DM: type 1 diabetes mellitus
T2DM: type 2 diabetes mellitus
