# Supplementary material for: Effectiveness of Mobile Health Interventions on Diabetes and Obesity Treatment and Management: Systematic Review of Systematic Reviews
Source: JMIR Mhealth Uhealth. 2020 Apr 28;8(4):e15400. doi: 10.2196/15400 (PMC7218595; doi:10.2196/15400)
Supplement: Multimedia Appendix 1 [file mhealth_v8i4e15400_app1.docx]

Appendix 1. Methodological quality of 17 studies based on AMSTAR2 criteria^a^

|  | Q1 | Q2 | Q3 | Q4 | Q5 | Q6 | Q7 | Q8 | Q9 | Q10 | Q11 | Q12 | Q13 | Q14 | Q15 | Q16 | Total |
| --- | --- | --- | --- | --- | --- | --- | --- | --- | --- | --- | --- | --- | --- | --- | --- | --- | --- |
| Wu et al., 2017[28] | Y | N | Y | Y | Y | Y | Y | Y | Y | N | Y | Y | Y | Y | Y | Y | 14 |
| Cui et al., 2016[27] | Y | Y | Y | Y | Y | Y | Y | Y | Y | N | Y | Y | Y | Y | Y | N | 14 |
| de Ridder et al., 2017[23] | Y | N | Y | Y | Y | N | Y | N | N | N | NA | NA | N | N | NA | Y | 6 |
| Kebede et al., 2017[32] | Y | Y | Y | Y | Y | Y | Y | Y | N | N | NA | NA | N | N | NA | Y | 9 |
| Hood et al., 2016[19] | Y | N | Y | N | Y | Y | Y | Y | N | N | NA | NA | N | N | NA | Y | 7 |
| Cotter et al., 2014[34] | Y | N | Y | N | Y | Y | Y | Y | N | N | NA | NA | N | N | NA | Y | 7 |
| Mallow et al., 2014[31] | Y | N | Y | N | N | N | N | Y | N | N | NA | NA | N | N | NA | Y | 4 |
| Baron et al., 2012[33] | Y | N | Y | Y | Y | Y | Y | Y | Y | N | NA | NA | Y | N | NA | N | 9 |
| Wang et al. 2017[21] | Y | Y | Y | N | N | Y | Y | Y | N | N | NA | NA | Y | Y | NA | N | 8 |
| Mateo et al., 2015[29] | Y | N | Y | Y | Y | Y | Y | Y | Y | N | Y | Y | Y | Y | Y | Y | 14 |
| Khokhar et al., 2014[30] | Y | Y | Y | Y | Y | Y | Y | Y | Y | N | Y | Y | Y | Y | Y | Y | 15 |
| Bhardwaj et al., 2017[22] | Y | Y | N | Y | Y | N | Y | Y | N | N | NA | NA | N | N | NA | N | 6 |
| Darling et al., 2017[36] | Y | Y | N | Y | N | N | Y | Y | Y | N | N | Y | Y | N | N | Y | 9 |
| Turner et al., 2015[35] | Y | Y | N | Y | Y | Y | Y | Y | Y | N | NA | NA | Y | N | NA | Y | 10 |
| Dobson et al., 2017 [50] | Y | Y | Y | Y | N | N | Y | Y | Y | N | NA | NA | Y | N | NA | Y | 9 |
| Wang et al., 2019 [51] | Y | Y | Y | Y | Y | Y | Y | Y | Y | N | Y | Y | Y | Y | Y | Y | 15 |
| Park et al., 2019 [52] | Y | Y | Y | Y | Y | Y | Y | Y | Y | N | Y | Y | Y | Y | Y | Y | 15 |

N: No, NA: Not able to answer, Y: Yes.

^a^AMSTAR 2 (Assessment of Multiple Systematic Reviews) is a modified AMSTAR criteria, used to assess the methodological quality of systematic reviews [53]. We assigned one point to each item that scored ‘yes’ and summed these to calculate a total score for each review. We classified quality of systemic reviews as high (score range: 12–16), moderate (score range: 9–11), low (score range: 5–8), or critically low (score range: 0–4) [54].
