# Supplementary material for: Effectiveness of Mobile Health Interventions on Diabetes and Obesity Treatment and Management: Systematic Review of Systematic Reviews
Source: JMIR Mhealth Uhealth. 2020 Apr 28;8(4):e15400. doi: 10.2196/15400 (PMC7218595; doi:10.2196/15400)
Supplement: Multimedia Appendix 2 [file mhealth_v8i4e15400_app2.docx]

Appendix 2. Characteristics of 17 review studies on the effectiveness of mobile health interventions for diabetes and obesity management.

| References^~~a~~^ | Study objective | Inclusion criteria | Search scope | | Intervention  tested | | Outcome measures | | | Meta-analysis |
| --- | --- | --- | --- | --- | --- | --- | --- | --- | --- | --- |
|  |  |  | Search database and date | Number of articles reviewed^b^ | Application type | Target function | (1) Clinical biomarkers | (2) Treatment adherence | (3) Behaviors | Outcomes |
| **I. Reviews on patients with diabetes** | | | | | | | | | | |
| Wang et al [21] | mHealth^c^ in type 1 diabetes mellitus management | RCT^d^, English | PubMed, Web of Science, EMBASE, etc; June 2016 | 3191, 424, and 8 | App and text messaging | Self-management | HbA_1c_^e^ | N/A^f^ | N/A | HbA_1c_ |
| Wu et al [22] | Diabetes self-management with apps | RCT | MEDLINE, EMBASE, CENTRAL, etc; January 2007-May 2016 | 1559, 145, and 96 | App | Self-management | HbA_1c_, severe hypoglycemia, and adverse effects | N/A | N/A | HbA_1c_ |
| III. **Reviews on patients with overweight/obesity** | | | | | | | | | | |
| Cui et al [23] | Diabetes self-management via apps | RCT | PubMed, Cochrane Library, and EMBASE; January 2005-June 2016 | 2596, 13, and 6 | App | Self- monitoring | HbA_1c_, body weight, blood glucose, BP^g^, and serum lipids | Glycemic self-control | N/A | HbA_1c_ |
| de Ridder et al [24] | Incentive-driven mHealth technology in diabetes management | English^h^ | IEEE, Springer, Science Direct, NCBI, etc; January 2008-August 2014 | 2182, 42, and 34 | App, text message, and internet | Education, reminder, feedback, social, and alert | HbA_1c_ and systolic BP | Glycemic self-monitoring | Diet and PA^i^ | N/A |
| Kebede et al [25] | Type 2 diabetes mellitus management with electronic health interventions | RCT,pre-post or quasi-experimental study, English^h^ | PubMed, Web of Science, and PsycINFO; January 1990-June 2016 | 1404, 227, and 32 | PDA^j^, text messaging, computer, and tablet | Self-management and education | HbA_1c_ and lipid profiles | Medication adherence, quantity of antidiabetic drugs, and use of drugs | Self-care tasks | N/A |
| Dobson et al [26] | Diabetes self-management | RCT, English | MEDLINE, PubMed, EMBASE, Cochrane Library, etc; January 2017 | 2368, 172, and 7 | Text messaging | Self-management | HbA_1c_ | Medication adherence and glycemic control | Diet, PA, self-care tasks, and knowledge | N/A |
| Hood et al [27] | Apps for diabetes self-management | English^h^ | PubMed, January 2016 | 485, N/A, and 13 | App | Self- management | HbA_1c_ and fasting blood glucose | N/A | N/A | N/A |
| Cotter et al [28] | Internet interventions for lifestyle modification | RCT/quasi-experimental study | PubMed, January 2013 | 2803, N/A, and 9 | App | Self-monitoring, education, and reminders | HbA_1c_, BMI, BP, and lipid levels | Glycemic control and medication adherence | Diet and PA | N/A |
| Mallow et al [29] | mHealth for rural diabetes care | RCT, English^h^ | PubMed, 2008-2012 | 157, 23, and 11 | App and text messaging | Self-monitoring and alerts | HbA_1c_, BP, cholesterol, and C-reactive protein | N/A | N/A | N/A |
| Baron et al [30] | Mobile monitoring on HbA_1c_ | Clinical trials, English^h^ | 6 electronic databases; August 2009-January 2012 | 8543, 104, and 24 (20 studies) | App | Self-monitoring, education, reminder, and feedback | HbA_1c_ | N/A | N/A | N/A |
| **II. Reviews on patients with obesity and diabetes** | | | | | | | | | | |
| Wang et al [31] | App interventions for obesity, diabetes treatment, and self- management | RCT/quasi-experimental study, English^h^ | PubMed, January 2000-August 2016 | 2129, 142, and 24 (obesity 14 and diabetes 10) | Apps, text messaging, PDA | Self-monitoring | Body weight and blood glucose | N/A | Obesity-related behaviors | N/A |
| Park et al, 2019 [32] | mHealth on weight loss management | RCT | MEDLINE, EMBASE, Cochrane Library, etc; October 2016 | 982, 121, and 20 | App and text messaging | Self-monitoring/management | Body weight and BMI | N/A | N/A | Body weight and BMI |
| Mateo et al [33] | Apps for weight loss and to increase PA | Clinical trials having a control group | PubMed, CINAHL, and Scopus; August 2015 | 946, 43, and 12 | App | Promoting weight loss and increase PA | Body weight and BMI | N/A | PA | Body weight, BMI, and PA |
| Khokhar et al  [34] | mHealth in weight loss among overweight/obese patients | RCT | Medline, PsycINFO, EMBASE, etc; May 2014 | 559, 108, and 6 | App, PDA, and text messaging | Self-monitoring, education, and counseling | Body weight and BMI | N/A | N/A | Body weight |
| Bhardwaj et al [35] | Management of adult obesity with mHealth | RCT, pilot, quasi-experimental, and descriptive design, English^h^ | PubMed, EBSCOhost, Google Scholar, etc; 2006-2016 | 5324, 80, and 54 | App, PDA, and text messaging | Self-monitoring, education or motivation, reminders, communication, and peer support | Body weight, waist circumference, and BMI | Adherence and satisfaction | PA and change in dietary behaviors | N/A |
| Darling et al [36] | Self-monitoring pediatric weight management via mHealth | RCT and pre-post, English^h^ | PubMed and PsycINFO; October 2016 | 16,355, 332, and 14 | App, text messaging, computer, PDA, and pedometer | Self-monitoring of pediatric weight | Weight status | N/A | Diet and PA | BMI, diet, and PA^k^ |
| Turner et al [37] | mHealth for prevention and treatment of pediatric obesity | RCT/pilot intervention studies, English^h^ | PubMed, Web of Science, EBSCOhost, etc; January-February 2014 | 4021, 41, and 18 | App, text messaging, etc | Education, entertainment, self-report, and feedback/ rewards | BMI | Adherence to treatment and self-monitoring | PA and breakfast/fruit/vegetable/ sugar-sweetened beverage consumption | N/A |

^a^Articles were ordered by topic, meta-analysis, and publication year.

^b^The number of papers indicated are of (1) studies accessed from the literature search, (2) full text screened for eligibility, and (3) studies included in the review, respectively.

^c^mHealth: mobile health.

^d^RCT: randomized controlled trial.

^e^HbA1_c_: hemoglobin A1_c_ (glycosylated hemoglobin).

^f^N/A: not available.

^g^BP: blood pressure.

^h^Some reviews had language inclusion criteria (English), but others did not clarify it.

^i^PA: physical activity.

^j^PDA: personal digital assistant, including a portable monitoring device.

^k^The meta-analysis included non-RCTs.

**REFERENCES**

21. Wang X, Shu W, Du J, Du M, Wang P, Xue M, Zheng H, Jiang Y, Yin S, Liang D, Wang R, Hou L. Mobile health in the management of type 1 diabetes: a systematic review and meta-analysis. BMC Endocr Disord. 2019;19(1):21.

22. Wu Y, Yao X, Vespasiani G, Nicolucci A, Dong Y, Kwong J, Li L, Sun X, Tian H, Li S. Mobile app-based interventions to support diabetes self-management: a systematic review of randomized controlled trials to identify functions associated with glycemic efficacy. JMIR mHealth uHealth; 2017;5(3):e35.

23. Cui M, Wu X, Mao J, Wang X, Nie M. T2DM self-management via smartphone applications: a systematic review and meta-analysis. PLoS One; 2016;11(11):e0166718.

24. de Ridder M, Kim J, Jing Y, Khadra M, Nanan R. A systematic review on incentive-driven mobile health technology: as used in diabetes management. J Telemed Telecare; 2017;23(1):26–35.

25. Kebede MM, Liedtke TP, Möllers T, Pischke CR. Characterizing Active Ingredients of eHealth Interventions Targeting Persons With Poorly Controlled Type 2 Diabetes Mellitus Using the Behavior Change Techniques Taxonomy: Scoping Review. J Med Internet Res. 2017;19(10):e348–e348.

26. Dobson R, Whittaker R, Pfaeffli Dale L, Maddison R. The effectiveness of text message-based self-management interventions for poorly-controlled diabetes: A systematic review. Digit Health. 2017;3:2055207617740315.

27. Hood M, Wilson R, Corsica J, Bradley L, Chirinos D, Vivo A. What do we know about mobile applications for diabetes self-management? A review of reviews. J Behav Med 2016;39(6):981–994.

28. Cotter AP, Durant N, Agne AA, Cherrington AL. Internet interventions to support lifestyle modification for diabetes management: a systematic review of the evidence. J Diabetes Complications. 2014;28(2):243–251.

29. Mallow JA, Theeke LA, Barnes ER, Whetsel T, Mallow BK. Using mHealth tools to improve rural diabetes care guided by the chronic care model. Online J Rural Nurs Heal care. 2014;14(1):43-65.

30. Baron J, McBain H, Newman S. The impact of mobile monitoring technologies on glycosylated hemoglobin in diabetes: a systematic review. J Diabetes Sci Technol. 2012;6(5):1185–1196.

31. Wang Y, Xue H, Huang Y, Huang L, Zhang D. A Systematic Review of Application and Effectiveness of mHealth Interventions for Obesity and Diabetes Treatment and Self-Management. Adv Nutr; 2017;8(3):449–462.

32. Park SH, Hwang J, Choi YK. Effect of Mobile Health on Obese Adults: A Systematic Review and Meta-Analysis. Healthc Inform Res. 2019;25(1):12-26.

33. Mateo GF, Granado-Font E, Ferré-Grau C, Montaña-Carreras X. Mobile phone apps to promote weight loss and increase physical activity: a systematic review and meta-analysis. J Med Internet Res; 2015;17(11):e253.

34. Khokhar B, Jones J, Ronksley PE, Armstrong MJ, Caird J, Rabi D. Effectiveness of mobile electronic devices in weight loss among overweight and obese populations: a systematic review and meta-analysis. BMC Obes; 2014;1(1):22.

35. Bhardwaj NN, Wodajo B, Gochipathala K, Paul III DP, Coustasse A. Can mHealth Revolutionize the Way We Manage Adult Obesity? Perspect Heal Inf Manag; 2017;14(Spring):1a.

36. Darling KE, Sato AF. Systematic Review and Meta-Analysis Examining the Effectiveness of Mobile Health Technologies in Using Self-Monitoring for Pediatric Weight Management. Child Obes. 2017;13(5):347–355.

37. Turner T, Spruijt‐Metz D, Wen CKF, Hingle MD. Prevention and treatment of pediatric obesity using mobile and wireless technologies: a systematic review. Pediatr Obes. 2015;10(6):403–409.
