# Supplementary material for: Effectiveness of Mobile Health Interventions on Diabetes and Obesity Treatment and Management: Systematic Review of Systematic Reviews
Source: JMIR Mhealth Uhealth. 2020 Apr 28;8(4):e15400. doi: 10.2196/15400 (PMC7218595; doi:10.2196/15400)
Supplement: Multimedia Appendix 3 [file mhealth_v8i4e15400_app3.docx]

Appendix 3. Summary of findings from the 17 reviews on the effectiveness of mobile health interventions for diabetes and obesity management.

| References^a^ | Tested interventions | Conclusion | Key results and health outcomes | | |
| --- | --- | --- | --- | --- | --- |
|  |  |  | (1) Clinical biomarkers | (2) Treatment adherence | (3) Behaviors |
| **I. Among patients with diabetes** | | | | | |
| Wang et al [21] | Self-management | mHealth^b^ may be effective in HbA_1c_^c^ care among patients with T1DM^d^ | The intervention group had a significant reduction in HbA_1c_ (−0.25, 95% CI −0.41 to −0.09) | N/A^e^ | N/A |
| de Ridder et al [24] | Education, reminder, feedback, social, and alert | Incentive-driven mHealth tools showed clinical effectiveness in some studies | HbA_1c_ levels were improved in 7 of 34 studies. Systolic BP^f^ was reduced in 2 of 34 studies | 13 of 34 studies had improved management practices— increased measurement frequency and complication checks | 13 of 34 studies had increased exercise and an improved diet |
| Kebede et al [25] | Self-management and education | mHealth interventions improved the level of HbA_1c_ | 25 of 32 studies reported a significant change in HbA_1c_ levels among persons with poorly controlled T2DM^g^ | N/A | N/A |
| Wu et al [22] | Self-management | Mobile app interventions improved glycemic control | Mobile app–based interventions were associated with a significant HbA_1c_ reduction of 0.48% and were larger for patients with T2DM than for patients with T1DM | N/A | N/A |
| Dobson et al [26] | Self-management | Text messaging for improvements in glycemic control in poorly controlled diabetes is mixed | 4 of 7 studies reported significant HbA_1c_ reduction in the intervention group | 2 of 3 studies reported significant improvements in blood glucose monitoring | 2 of 5 studies reported significant improvements in PA^h^, exercise, or diet |
| Cui et al [23] | Self- monitoring | Smartphone apps offered a moderate effect on glycemic control | HbA_1c_: using apps had 0.4% lower reduction than the control group (*P*=.007).  0.3% reduction among individuals with baseline HbA_1c_ of <8%. No effects on BP, serum lipids, or weight | A moderate effect on glycemic control | N/A |
| Hood et al [27] | Self-management | Mobile apps are promising for diabetes self-management | 4 of 13 studies reported significant blood glucose level improvement | N/A | N/A |
| Cotter et al [28] | Self-monitoring, education, and reminders | mHealth strategies are a feasible option for diabetes self-management | HbA_1c_: 2 of 7 studies reported improvement. BMI: 1 of 4 studies reported a decrease in BMI. BP: none of the 4 studies reported a decrease. Lipid: none of the 5 studies reported a decrease | 1 of 9 studies reported improvement in self-reported glucose monitoring. None of the 2 studies reported changes in medication adherence | Eating habits and fat intake improvements—25% of studies; PA improvements—40% of studies |
| Mallow et al [29] | Self-monitoring, alerts, and messaging | mHealth has the potential to improve health outcomes in rural underserved populations | 9 of 11 studies reported a significant reduction in HbA_1c_, 3 studies reported decreasing BP, and 1 study reported improved cholesterol and C-reactive protein levels | N/A | N/A |
| Baron et al [30] | Self-monitoring | The effectiveness of mHealth interventions was inconsistent and weak | 3 of 7 studies reported significant improvements in HbA_1c_ levels among patients with T1DM and 10 of 13 studies reported significant improvements in HbA1c levels among patients with T2DM | N/A | N/A |
| **II. Among patients with diabetes and obesity** | | | | | |
| Wang et al [31] | Self-monitoring | Some mHealth interventions were effective and promising | 6 of 14 studies reported weight loss or a decrease in waist circumference—weight loss from −2 kg in 16 weeks to −7 kg in 5 weeks; 5 of 10 studies for diabetes management reported reduced HbA_1c_ and blood glucose levels—HbA_1c_ from −0.4% in 10 months to −1.9% in 12 months | N/A | 5 of 14 studies for obesity management reported behavior changes. No behavior changes were observed in diabetes studies |
| **III. Among patients with overweight or obesity** | | | | | |
| Park et al [32] | Self-monitoring and management | Use of mHealth for obese adults showed a modest short-term effect on body weight and BMI | Body weight was significantly reduced with a weighted mean difference of −2.35 kg (95% CI −2.84 to −1.87) | N/A | N/A |
| Bhardwaj et al [35] | Education or motivation, reminders, communication, self-monitoring, and peer support | Most mHealth interventions had a favorable influence on weight reduction and behavior changes | 9 of 14 studies reported better body weight changes. 3 studies reported a reduction in waist circumference.  3 of 5 studies reported a decrease in BMI | 10 studies reported better adherence in intervention groups versus control groups, ranging from 60%-85% | 6 studies found changes in eating behaviors (calories, vegetable, and fruit). 5 of 9 studies found PA or exercise levels increased in intervention versus control groups |
| Darling et al [36] | Self-monitoring of pediatric weight | mHealth self-monitoring had less benefits on children’s weight status and diet | Weight reduction after the intervention was less (mean difference 0.42, 95%CI −0.66 to −0.19) | N/A | Dietary intake (sugar-sweetened beverages, fruit, and vegetable) after the intervention was improved (mean difference score 0.10, 95% CI 0.02 to 0.24), but no significant change in PA (mean difference score 0.48, 95% CI −0.32 to 1.29) |
| Mateo et al [33] | Promoting weight loss and increase in PA | mHealth may be a useful tool in weight loss | The intervention group had a higher decrease: mean difference in body weight change −1.04 kg (95% CI −1.75 to −0.34) in 9 trials; mean difference in BMI change −0.43 kg/m^2^ (95% CI −0.74 to −0.13) in 8 trials | N/A | No significant improvement in PA was shown in 7 trials |
| Turner et al [37] | Education, entertainment, self-report, and feedback or rewards | mHealth approaches are feasible in the prevention and treatment of pediatric obesity | Few studies found significant changes in BMI after the intervention | Self-monitoring, goal setting and tracking, and adherence to interventions increased, whereas attrition rate reduced | Frequency of breakfast, fruit, and vegetable intake and PA increased. No significant change in sugar-sweetened beverage intake |
| Khokhar et al [34] | Self-monitoring, education, and counseling | mHealth had a potential to facilitate weight loss in patients with overweight and obesity | Intervention groups using a mobile device had a higher body weight reduction (−1.09 kg, 95% CI −2.12 to −0.05) than control groups.  Using mobile phones was more effective in achieving weight loss than other devices (−1.78 kg, 95% CI −2.92 to −0.63) | N/A | N/A |

^a^Articles were ordered by topic and publication year.

^b^mHealth: mobile health.

^c^HbA_1c_: hemoglobin A_1c_ (glycosylated hemoglobin).

^d^T1DM: type 1 diabetes mellitus.

^e^N/A: not available.

^f^BP: blood pressure.

^g^T2DM: type 2 diabetes mellitus.

^h^PA: physical activity.

**REFERENCES**

21. Wang X, Shu W, Du J, Du M, Wang P, Xue M, Zheng H, Jiang Y, Yin S, Liang D, Wang R, Hou L. Mobile health in the management of type 1 diabetes: a systematic review and meta-analysis. BMC Endocr Disord. 2019;19(1):21.

22. Wu Y, Yao X, Vespasiani G, Nicolucci A, Dong Y, Kwong J, Li L, Sun X, Tian H, Li S. Mobile app-based interventions to support diabetes self-management: a systematic review of randomized controlled trials to identify functions associated with glycemic efficacy. JMIR mHealth uHealth; 2017;5(3):e35.

23. Cui M, Wu X, Mao J, Wang X, Nie M. T2DM self-management via smartphone applications: a systematic review and meta-analysis. PLoS One; 2016;11(11):e0166718.

24. de Ridder M, Kim J, Jing Y, Khadra M, Nanan R. A systematic review on incentive-driven mobile health technology: as used in diabetes management. J Telemed Telecare; 2017;23(1):26–35.

25. Kebede MM, Liedtke TP, Möllers T, Pischke CR. Characterizing Active Ingredients of eHealth Interventions Targeting Persons With Poorly Controlled Type 2 Diabetes Mellitus Using the Behavior Change Techniques Taxonomy: Scoping Review. J Med Internet Res. 2017;19(10):e348–e348.

26. Dobson R, Whittaker R, Pfaeffli Dale L, Maddison R. The effectiveness of text message-based self-management interventions for poorly-controlled diabetes: A systematic review. Digit Health. 2017;3:2055207617740315.

27. Hood M, Wilson R, Corsica J, Bradley L, Chirinos D, Vivo A. What do we know about mobile applications for diabetes self-management? A review of reviews. J Behav Med 2016;39(6):981–994.

28. Cotter AP, Durant N, Agne AA, Cherrington AL. Internet interventions to support lifestyle modification for diabetes management: a systematic review of the evidence. J Diabetes Complications. 2014;28(2):243–251.

29. Mallow JA, Theeke LA, Barnes ER, Whetsel T, Mallow BK. Using mHealth tools to improve rural diabetes care guided by the chronic care model. Online J Rural Nurs Heal care. 2014;14(1):43-65.

30. Baron J, McBain H, Newman S. The impact of mobile monitoring technologies on glycosylated hemoglobin in diabetes: a systematic review. J Diabetes Sci Technol. 2012;6(5):1185–1196.

31. Wang Y, Xue H, Huang Y, Huang L, Zhang D. A Systematic Review of Application and Effectiveness of mHealth Interventions for Obesity and Diabetes Treatment and Self-Management. Adv Nutr; 2017;8(3):449–462.

32. Park SH, Hwang J, Choi YK. Effect of Mobile Health on Obese Adults: A Systematic Review and Meta-Analysis. Healthc Inform Res. 2019;25(1):12-26.

33. Mateo GF, Granado-Font E, Ferré-Grau C, Montaña-Carreras X. Mobile phone apps to promote weight loss and increase physical activity: a systematic review and meta-analysis. J Med Internet Res; 2015;17(11):e253.

34. Khokhar B, Jones J, Ronksley PE, Armstrong MJ, Caird J, Rabi D. Effectiveness of mobile electronic devices in weight loss among overweight and obese populations: a systematic review and meta-analysis. BMC Obes; 2014;1(1):22.

35. Bhardwaj NN, Wodajo B, Gochipathala K, Paul III DP, Coustasse A. Can mHealth Revolutionize the Way We Manage Adult Obesity? Perspect Heal Inf Manag; 2017;14(Spring):1a.

36. Darling KE, Sato AF. Systematic Review and Meta-Analysis Examining the Effectiveness of Mobile Health Technologies in Using Self-Monitoring for Pediatric Weight Management. Child Obes. 2017;13(5):347–355.

37. Turner T, Spruijt‐Metz D, Wen CKF, Hingle MD. Prevention and treatment of pediatric obesity using mobile and wireless technologies: a systematic review. Pediatr Obes. 2015;10(6):403–409. 38.
